# Are multifunctional marine polysaccharides a myth or reality?

**DOI:** 10.3389/fchem.2015.00039

**Published:** 2015-06-30

**Authors:** Svetlana Ermakova, Mikhail Kusaykin, Antonio Trincone, Zvyagintseva Tatiana

**Affiliations:** ^1^G.B. Elyakov Pacific Institute of Bioorganic Chemistry, Far Eastern Branch of the Russian Academy of SciencesVladivostok, Russia; ^2^Istituto di Chimica Biomolecolare, Consiglio Nazionale delle RicerchePozzuoli, Italy

**Keywords:** brown algae, polysaccharide, fucoidan, fucoidanase, food supplement, biological activities

Marine algae are ancient photosynthetic organisms that constitute the largest group in the plant kingdom. They are used for functional food, cosmetic additives, supplements productions, and in traditional medicine due to taste, prophylactic, and therapeutic effects. Algae contain microelements and iodine-containing organic compounds, as well as vitamins, mannitol more than terrestrial plants.

Polysaccharides of algae are especially valuable substances. Some of them (for example agarose, carrageenans, and alginates) have found widespread application. Information about them was published a long time ago and described in detail in books. At the moment polysaccharides synthesized by brown algae (laminarans and especially fucoidans) are of greatest interest. A laminarans were found in both marine and terrestrial organisms. It should be noticed that fucoidans are truly marine polysaccharides. The general term “fucoidan” is used to integrate the molecules, differenced in composition, structure, and in degree of sulfation, acetylation, etc. (Berteau and Mulloy, 2003; Kusaykin et al., 2008). Content of fucoidans depends on the species and on the stage of development of algae and may vary from 0.1 to 20% of dry weight of algae (Mabeau et al., 1990; Zvyagintseva et al., 2003). Huge amount of reserves of fucoidans accumulate in brown algae, which grow in the seas at temperate and northern latitudes (Ermakova et al., 2011; Sokolova et al., 2011; Men'shova et al., 2012; Thinh et al., 2013). Analogs of these polysaccharides have not been found on the land till now. Fucoidans long since are attracted attention due to diverse biological activity, low toxicity, and plant origin (Berteau and Mulloy, 2003; Kusaykin et al., 2008). Last is important because of contamination and side effects of the preparation produced from animals (for example, heparin).

A large number of publications are devoted to the study of antitumor, anticoagulant, antimutagenic activities, and immunostimulatory, antiinfective and antioxidant properties of these polysaccharides. However, despite the obvious prospects for exploitation in medicine, none of fucoidan is declared yet as a drug. The reason is that the structural diversity of fucoidans is extremely large. Structural investigation of fucoidans is of great difficulties because of varieties of monosaccharide compositions, different types of glycosidic linkages, presence of large numbers of non-carbohydrate substituents. There are only a small number of fucoidans with established basic elements of the chemical structure (Chizhov et al., 1999; Bilan et al., 2002, 2004, 2008; Zvyagintseva et al., 2003; Shevchenko et al., 2007; Anastyuk et al., 2009, 2010, 2014; Kuznetsova, 2009; Ale et al., 2011; Vishchuk et al., 2011, 2013; Thinh et al., 2013). Unfortunately in the study of biological properties and enzymatic transformations of these molecules, fucoidans with unidentifiable structure are often used, thus reducing the generalization of the results obtained. Over the past 15–20 years there has been an increase in the number of structural studies of fucoidans. It became obvious that the study of their biological action, without regard to the structure does not allow to create drugs based on these polysaccharides.

It is now considered that fucoidans are species-specific polysaccharides. This means that each alga synthesizes fucoidan or set of fucoidans characteristic only for it. In monosaccharide composition of fucoidans necessarily there are sulfated residues of fucose and often galactose. As minor components residues of mannose, glucuronic acid, xylose, and other more rare monosaccharides, are present (Kusaykin et al., 2008).

1,3-α-L-Fucans are most often found in algae (Zvyagintseva et al., 2003; Anastyuk et al., 2010). α-1,4-Glycosidic linkage between L-fucose residues is less common and is present mainly as a 1,3;1,4-α-L-fucans. Brown algae also often synthesize galactofucans. The position and content of galactose residues in various galactofucans depend on the type of algae; content is frequently comparable to the that of fucose (Shevchenko et al., 2007; Anastyuk et al., 2009; Thinh et al., 2013). This is the most structurally diverse group of fucoidans. A smallest group of fucoidans is represented by fucomannuronans (Imbs et al., 2011). Furthermore, there are fucoidans, containing more heterogeneous monosaccharide composition.

In order to establish the structure of polysaccharides the most promising approach is based on the use of enzymes. Enzymatic transformation of polysaccharides can be extremely useful not only for the establishment of structural features, but also for the access to biologically active fragments (Silchenko et al., 2013; Menshova et al., 2014; Trincone, 2014). Reports about producers and properties of the enzymes (fucoidanases) are rare despite the growing interest in the fucoidans (Kusaykin et al., 2008). No more than 20 producers of fucoidanases are known, mainly isolated from marine fungi and bacteria (Sakai et al., 2003; Descamps et al., 2006; Rodriguez-Jasso et al., 2010; Silchenko et al., 2013, 2014). This rareness is due to the absence of quantitative simple methods for determination of the activity of fucoidanases. Precise assessment of enzymatic features is also hampered by the use of structurally uncharacterized substrates. So, in their transformation enzymes with different specificities should be involved.

Few sources of fucoidanases were found among marine invertebrates (Kitamura et al., 1992; Giordano et al., 2006; Silchenko et al., 2014). Fucoidanase in *Patinopecten yessoensis* was discovered in 1992 by the action on fucoidan from *Nemacystus decipiens* (Kitamura et al., 1992). Information about the structure of substrate reported in the article, consisting of L-fucose residues and small amounts of D-galactose residues, is quite scarce. Data about the type of glycosidic linkages are absent (Tako et al., 1999). High molecular weight products (about 50 kDa) formed sufficiently under the action of fucoidanase from *P. yessoensis*. Information about their structures is not available.

We found new sources of fucoidanases: the vietnamese mollusk *Lambis* sp. and the marine bacteria *Formosa algae* KMM 3553 (Khanh et al., 2011; Silchenko et al., 2014). Analysis of the hydrolysis products of fucoidans with established structure from collection of our laboratory, showed that both fucoidanases are endo-enzymes hydrolyzing α-1,4-glycosidic linkages in fucans (Silchenko et al., 2014).

Purification grade of fucoidans is also important for the investigation of biological properties. Unfortunately, uncharacterized crude preparations are often used even in scientific research. Methods for isolation and purification of fucoidan may be different. The most universal scheme includes preprocessing of algae by organic solvents extracting most secondary metabolites, such as polyphenols and other UV absorbing compounds (Shevchenko et al., 2005). These substances, usually powerful antioxidants, often are strongly associated with fucoidans and removal of them entails great difficulties. We show that the purification of fucoidans from impurities results in a loss of antioxidant activity (Imbs et al., 2015). Not only antioxidant, but also antibacterial activity of fucoidans can be completely or partially due to impurities. Separation of fucoidans from them is not always possible, as polysaccharides often form strong complexes with polyphenols, which cannot be destroyed without affecting the integrity of the fucoidan molecules. Nevertheless the evidence of antioxidant activity due to impurities of fucoidans were studied quite intensively (Wang et al., 2008; Hu et al., 2010; Costa et al., 2011). However the data on the purity of fucoidans is often absent.

It is interesting to note that specific biological activities of fucoidans are associated with their structures. So, the formation and growth of the colony of breast cancer cells are suppressed by galactofucans from *Saccharina japonica* and *Undaria pinnatifida*. Human colon cancer cells are more sensitive to fucoidan from *Saccharina cichorioides* (consisted of (1→3)-α-L-fucose residues), human melanoma cells—to fucoidan from *Fucus evanescens* (Moon et al., 2009; Vishchuk et al., 2011, 2013).

Thus, the intensification of structural studies of fucoidans and the use of highly purified preparations will help to dispel some myths about the effect of fucoidans on organisms and to outline the range of biological properties only related to polysaccharides. The first is immunomodulatory (Khil'chenko et al., 2011), antibacterial, antiviral (Prokofjeva et al., 2013), and antitumor activities (Ermakova et al., 2011; Vishchuk et al., 2011, 2013).

In Russia in 2006 the suplement “Fucolam®” (No 77.99.23.3.y.739.1.06, Russia), based on structurally characterized fucoidan from the brown alga *Fucus evanescens*, synthesizing from 12 to 15% of the polysaccharide, was registered. The biological effects of the “Fucolam®” are studied in detail. It was established that the “Fucolam®” in addition to the immunomodulatory, antibacterial, antiviral, and antitumoral activities has probiotic, hepatoprotective, glucose, and cholesterol lowering effects (Drozd et al., 2006, 2011; Kuznetsova, 2009; Khil'chenko et al., 2011; Lapikova et al., 2012; Besednova et al., 2014, 2015; Zaporozhets et al., 2014). It is a prominent representative of multifunctional agent and can serve as the base for drug development.

According to known data from the studies above mentioned, the spectrum of biological properties of fucoidans is wide enough. These natural substances are outstanding representatives of multifunctional compounds, and this is not a myth but a reality.

## Conflict of interest statement

The authors declare that the research was conducted in the absence of any commercial or financial relationships that could be construed as a potential conflict of interest.
